# Beyond the Benefits of Assistance Dogs: Exploring Challenges Experienced by First-Time Handlers

**DOI:** 10.3390/ani9050203

**Published:** 2019-04-29

**Authors:** Jennifer Gravrok, Dan Bendrups, Tiffani Howell, Pauleen Bennett

**Affiliations:** 1 Anthrozoology Research Group, School of Psychology and Public Health, La Trobe University, Edwards Rd, Flora Hill, VIC 3552, Australia; t.howell@latrobe.edu.au (T.H.); pauleen.bennett@latrobe.edu.au (P.B.); 2 Research Education and Development Team, Graduate Research School, La Trobe University, Edwards Rd, Flora Hill, VIC 3552, Australia; d.bendrups@latrobe.edu.au

**Keywords:** service dogs, disability, thriving, assistive technology

## Abstract

**Simple Summary:**

People with disabilities employ many forms of assistive technology, including assistance dogs (AD), to assist them with managing their disability. Most previous research has focused on the benefits of ADs for their handlers with disability; little is known about the challenges they face. The aim of this study was to explore the experiences of first-time handlers when working with an AD. It was found that handlers experienced many benefits from their dog, as would be expected. However, they also reported experiencing many challenges which hindered or delayed these benefits. These challenges arose from the handler’s medical conditions, cognitive ability and social environment, as well as from dog-related factors. They are important for potential handlers and AD organizations to consider prior to placing an AD, since this will assist individuals and organizations to better determine if an AD is the right form of assistive technology for a particular individual, and, if so, how best to prepare to integrate the dog into the person’s life.

**Abstract:**

The purpose of this study was to explore first time handlers’ experiences when working with an assistance dog (AD). Interviewees included seven first time AD handlers and 14 other individuals close to these handlers, including family members, carers and AD instructors. Semi-structured interviews were conducted six months and one year after each handler received their AD. Interview questions were informed by the Thriving Through Relationships theory of social support and previous interviews with the participants. Inductive content analysis corroborated previous findings regarding the benefits that ADs provide. In addition, four factors were revealed to substantially influence the challenges handlers experienced when learning to utilize their dog. These included the handlers’ medical conditions, cognitive ability and social environment, and dog-related factors. Organizations would benefit from considering these factors in their operational processes.

## 1. Introduction

People with disabilities, especially chronic disabilities, often face high levels of adversity and have pervasive support needs that encompass many life domains [1]. Multiple types of support are available to ease challenges associated with specific disabilities, including assistive technology or recruitment of carers [2,3]. The implementation of support may affect an individual’s self-perception and ultimately impact their well-being or quality of life [4].

An assistance dog (AD) is one form of assistive technology, with these dogs being trained to provide disability-specific support to one person (the handler) who has a disability or disabilities [4,5]. Functions these dogs are trained to perform typically include physical tasks, which provide performance benefits [6]. In addition, ADs are reported to provide dynamic emotional, social and psychological benefits [7], which increase the handler’s wellbeing [8] or quality of life [9]. Handlers also experience less negative stigma than is commonly associated with other forms of support [10]. 

The benefits ADs are known to provide have been repeatedly and extensively reported in the literature [11,12]. However, the prevailing research focus is on benefits and thus minimizes the information available about challenges people experience. Previous work has demonstrated that many challenges were expected prior to acquiring an AD [13] and experienced when handlers began to work with their AD [14]. Other researchers have briefly mentioned challenges experienced at later time points as well [15]. Therefore, developing a holistic understanding of experiences handlers have with an AD is important. 

The purpose of this paper is to document challenges experienced when working with an AD. To accomplish this, semi-structured interviews were conducted with first-time AD handlers and other individuals who have a significant impact on the handler’s life. Although many benefits were reported, information about challenges was selectively extracted from the interview data. This enabled better understanding of how various factors contribute to the challenges that AD handlers experience.

## 2. Materials and Methods

La Trobe University Human ethics committee approved all procedures (HEC16-106).

### 2.1. Participants

First-time AD handlers were recruited from three AD organizations in Australia for voluntary participation in this study. Seven handlers volunteered and formed the basis of seven case studies. The handler could nominate other individuals to participate as well. Nominated individuals had a relationship with the handler and dog, but each provided a different perspective on the handler-dog relationship. Individuals, as can be seen in Table 1, included: parents (*n* = 6), carers/others (*n* = 3) and AD instructors (*n* = 5). Instructors were people employed by the AD organizations to teach the handler to work with their dog. 

### 2.2. Materials

Two elements of a multi-part study [13,14] preceded the final component of the investigation, which is reported in this paper. Semi-structured interviews were conducted by JG. Previous information collected from these individuals enabled personalization of the interview questions. The Thriving Through Relationships theory of social support [16,17,18,19] provided the conceptual underpinning for the interview questions. This theory has been shown to be potentially relevant to the AD context [20,21]. The initial questions were only loosely specified, however, so this enabled the interviewer to elicit more nuanced responses when appropriate. 

### 2.3. Procedures

Interviews were conducted between October 2017 and February 2019, approximately 6 and 12 months after each handler received their dog. Written informed consent was obtained for all participants prior to the interview; child participants provided verbal assent, along with parental written consent. Interviews ranged from 30 to 60 min. The timeline of interviews is presented in Table 1, along with the mode of interview. In-person interviews were conducted at a location familiar to the interviewee. Some handlers were interviewed with a nominated individual who knew them well in attendance. For handlers who experienced intellectual disabilities or speech impediments, this strategy facilitated a continuation of conversation and enhanced understanding. 

### 2.4. Analysis

All interviews were audio recorded, transcribed, de-identified, and uploaded to QSR International’s NVivo 10 qualitative data analysis software, by JG. Inductive content analysis was conducted [22] by JG and validated through extensive discussions with other members of the research team. Due to the various perspectives included, data source triangulation enhanced the reliability of the findings [23,24]. De-identified transcripts are available from the authors upon request, but original data cannot be made available due to the identifiable nature of this material. 

## 3. Results

The analysis revealed that most of the information collected from the interviews confirmed already established benefits that ADs provide [12]. Rather than focusing on these previously reported benefits, the focus in this paper is on the portion of the data that illuminates complexities experienced when working with an AD. This emphasis will contribute to building a more holistic understanding of what handlers and their social contacts experience when working with an AD. 

Inductive content analysis revealed that four main factors contribute to the challenges experienced when working with an AD. These were: the medical condition/s experienced by the handler, their cognitive ability, their social environment, and dog-related factors. For some handlers, these factors were so problematic that they substantially reduced the benefits the handler was able to receive from their AD. Each factor is briefly discussed below, with quotes from participants illustrating negative impacts when these were apparent. 

### 3.1. Medical Condition

Handlers who experienced multiple or complex medical conditions, particularly if these consistently or rapidly changed, experienced more challenges compared to people with single or relatively constant disabilities. These challenges were derived from the changing conditions of the disabilities themselves, but also from associated mental health challenges and extended hospital stays. 

#### 3.1.1. Changing Medical Conditions

The nature of the medical condition, such as its complexity and stability, was a significant factor in determining the benefits received from the AD. For example, for some participants, certain times of the year were more challenging for them medically. As one mother stated:

“the difficulty we have at this time of year is that [H6] has autonomic difficulties and she can’t cope with the heat at all. So basically, she goes from house to car to shopping center, or an appointment. She can’t go for a walk on the street”.

(P6)

This led to challenges for the handler in performing all the functions necessary to care for their dog. Consequently, the dog bonded more than was desired with other family members, who were required to perform these functions, and benefits such as increased independence and exercise were not received by the handler. 

Individuals whose physical and mental health fluctuated markedly from day to day also experienced extreme challenges in consistency and developing rules. One instructor described:

“we had so many surprises in this program, they [H4 and the family] go away, they come back, today he [the handler] is not well, tomorrow he is. They had so many issues with school and so many issues with this and that […] in this program [there are] almost no rules, we make a rule and then we need to change it as something else happens”.

(ADI4)

This slowed training progression and integration of the dog into the handler’s life, which consequently delayed the training for the dog to alert to the handler’s medical condition. 

Another handler carefully described how she believed her complex medical condition created more challenges than those faced by individuals with less complex disabilities:

“I think it’s really difficult because with ADs, a lot of it is about your specific disability rather than the dog. Like whether things work out or not because if you have, I’m careful with my words here, something a little less complex, like you are blind or you have diabetes where the dog has one job and things were a little more predictable in your everyday life, then I think things are a lot easier. But, because my health is one day at a time sometimes, and there’s so many factors, like fatigue and seizures and mobility and she’s got so many tasks and so many jobs that she is meant to be doing all at the same time, she’s really not just an alert dog, she’s not just a mobility dog, she’s not just a psychiatric AD, she is ALL of those things combined”.

(H6)

The complex nature of this handler’s disabilities slowed training progression and, therefore, she did not receive benefits such as confidence and independence as early or as consistently as handlers with less complex disabilities. 

#### 3.1.2. Mental Health

A majority of handlers experienced mental health challenges. For some handlers, their mental health significantly impacted their concentration, tiredness, stress, motivation and assertiveness. For one handler, this meant that she could not reinforce the training:

“A lot of the time I don’t have the energy to follow through and be as consistent with the house training. So, if I call her and she doesn’t come straight away a lot of the time I don’t have the [energy] to follow through and actually say ‘[dog] come’”.

(H6)

This allowed the dog to learn that she could get away with undesirable behaviors. As a result, the dog was required to return to the training organization for one month to fix these behavior problems. For others, their ability to maintain control changed from day to day depending on their mental health. For one handler who received considerable benefits from companionship with his dog, this was noted to decrease when he was experiencing mental health issues. His carer noted: “I think as a companion it [the dog] has de-stressed him when the mental health issues haven’t been an issue. When the mental health issues are an issue, I don’t think [the dog] has had any major effect on it.” (O2). This AD provided considerable companionship and socialization benefits to the handler most days, however this was minimized when mental health issues were prevalent. Overall, participants emphasized how much the fluctuation in mental health impacted the handler-dog relationship, contributing to regression in the dog’s abilities. This reduced the overall benefits obtained but also sometimes created welfare issues for the dog, and a subsequent need for an extended period of rehabilitation/re-training. 

#### 3.1.3. Hospital Admissions

Three participants had extended hospital admissions within the first year with their dog. Two of these handlers were not able to keep their dog in the hospital with them for the extended period. This was due to the handlers’ inability to care for and toilet the dog while in hospital. Even the handler who kept the dog with him during the hospital admission noted a regression in training. One participant noted: “we did feel that [the hospitalizations] probably had slowed the process in terms of bonding and then achieving the goals, in terms of being more independent and probably the alerting behavior might be a little sharper by now” (O6). Hospital admissions greatly impacted bonding and the handler’s ability to thrive, as stated by one mother: “She spent 163 nights in hospital last year, she is not thriving. She’s not well. Maybe she would be less thriving without [the dog], but she is certainly not thriving” (P6). Hospital admissions were unavoidable and hindered the receipt of many benefits as the dog could not be physically present. 

### 3.2. Cognitive Ability

Some handlers’ medical conditions affected their cognitive ability, which negatively impacted some individuals’ ability to receive benefits from their dog. Compared to those with adult-level cognitive abilities, handlers who had an intellectual disability or who lacked maturity (due to age) experienced more challenges related to memory and consistency in handling the dog and thus maintaining its trained behavior. 

#### 3.2.1. Maturity

Lack of maturity was identified in two young handlers, where it contributed to a deficiency in forward thinking or an inability to think outside themselves. While considered normal for their age, this was not conducive to taking care of their dog. One instructor noted that the handler: “doesn’t have any perception of forward thinking in how this is going to relate to her. Her world is, I am starting to find out with kids, is just their body, anything outside of arm’s reach is not there” (ADI7). This impacted the handlers’ motivation to take care of their dog, which weakened the bond and companionship received, and potentially adversely affected the dog. Additionally, after placement it was realized that one handler lacked many of the prerequisite skills to work with an AD. The lack of skill was partly due to maturity, as the handler had not reached a high level of skills to manage her disability due to her young age. Although she was able to increase independence around school, this considerably decreased the overall amount of independence she gained. 

#### 3.2.2. Intellectual Disability

One handler had minor intellectual disability, which caused challenges in independently remembering to take care of the dog. Another handler experienced more severe intellectual disability, which caused many unforeseen challenges, such as lack of concentration, sending mixed signals to the dog, and lack of ability to retain knowledge about dogs. A lack of ability to concentrate on more than one task at a time hindered the handler and the dog’s safety. One carer noted: “He will be so concentrating on what he has to do with the dog that he will forget about road safety and things like that” (O2). Therefore, this handler always needed a carer with him. Support from additional carers and lack of assertiveness compared to a carer when delivering commands caused the dog to receive mixed signals regarding who the main care provider was. This was undesirable as ADs should primarily rely on the handler. It was also clear that the handler who experienced more intellectual disability lacked basic knowledge about dogs even one year after living with one and two years after working with an AD through the organization’s training program. During the final interview for example, the handler spontaneously asked how to tell if a dog was relieving itself or when to give the dog treats. 

In both situations, where handlers lacked maturity due to age or experienced intellectual disability, the welfare of the dog was often maintained by a parent or carer being involved. Although this was necessary for the dog, this potentially delayed the AD-handler bond. Additionally, this involvement by other individuals hindered the amount of responsibility and independence the handler could gain from having the AD.

### 3.3. Social Environment

Most participants emphasized the impact the social environment and social support had on the success of the dog: “It’s a real community that needs to come together to work for this child, for them to live a full life. If you don’t have that from each direction, from each person that is involved, then it’s not as successful” (O7). When this support was not available, the handler experienced more challenges. Social environments in group homes or community day programs and relationships with carers caused the most challenges. 

#### 3.3.1. Group Home and Community Day Program Environment

Group homes and community day programs caused major hindrances that delayed much of the bonding and integration of the dog into the handler’s daily life. These organizations had many concerns that they wanted addressed before they allowed an AD to attend:

“Their main concerns are OHAS [occupational health and safety], tripping hazards, [the dog] getting hurt by anybody that’s having some behavioral issues. They are concerned that there are some people there that might have a fear of dogs, so they are very concerned about upsetting and causing any extra stress”.

(ADI2)

These concerns were valid, but existed primarily because none of these organizations had experience with a client having an AD previously. One such organization was also hesitant because they did not understand the impact that the dog could have on the handler’s life: “they are sort of like ‘what is [the dog] here for? [H2] has been coming for seven years, he hasn’t needed a dog before, why does he need one now?’” (P2). This slowed integration of the dog into the handler’s life, delaying many benefits. Additionally, as handlers who attended these locations had intellectual disabilities, they required assistance from the staff. Therefore, training the care staff to properly assist the handler with the AD was a challenge because the care staff was constantly changing, making it impossible to educate everyone who interacted with the handler on how to work with the dog. This created inconsistency in the training and led to the development of undesirable behaviours in the dog, which did not promote confidence in the dog with facilities that were initially hesitant to include the AD. This subsequently delayed socialization and companionship benefits the handler could receive. 

#### 3.3.2. Relationships with Carers

The relationships handlers had with certain carers caused many challenges, especially for those handlers who experienced cognitive challenges. Some of the challenges were due to the carers’ lack of understanding or training regarding ADs. Often carers had personal beliefs about how dogs should be treated or trained, which differed from how ADs are trained. They often did not understand the reasons for the AD rules and therefore lacked consistency or failed to maintain the rules. Personal beliefs also impacted the handler-carer relationship. For example, one mother explained: “there was a guy from another organization that used to take [H2] out every Saturday. Soon as we said there was going to be a dog he said he wouldn’t have the dog in the car” (P2). Consequently, this relationship ended. Individuals without cognitive impairments experienced far fewer challenges with carers regarding their dog. Although they may have carers for physical assistance, they were typically less involved in the care or support of the dog. 

### 3.4. Dog Factors

Some dogs themselves also caused some concern, primarily through their inability to reliably perform the main function they were acquired for, such as alerting behavior, and immaturity at the time of initial placement. 

#### 3.4.1. Inability of the Dog to Alert

Three of the dogs were acquired to work as medical or seizure alert dogs. Of these, one dog was still not trained to alert to the medical condition at six months post-placement, and the other two dogs were trained but were not alerting reliably after one year of working with the handler. Individuals involved in both case studies recognized that the dog’s ability to respond to a medical event was more reliable than their ability to alert prior to the event. One mother described this as:

“We are more likely to pick up on [a seizure] than [the dog] is, and then what she does it, she responds to our behavior. She sees us going ‘oh are you okay?’ then she is like OH! Then she barks. So, it’s delayed and it’s reacting to our behaviors”.

(P1)

This was reported to occur in instances where the dog was out of sight of the handler (e.g., under a table). According to the instructors, however, this should not impact the dog’s ability to alert. 

The consequences of the dog not being able to alert reliably was that the handler’s safety was potentially compromised. At the time of their interviews, all handlers had gained sufficient confidence to walk independently in their neighborhood with their dog. While this is a clear benefit of having an AD, if a medical event was to happen because the dog did not alert, the handler may be in more danger than previously as they may be further from a knowledgeable responsive individual. “She’s not alerting before episodes, and I still am walking sometimes and like wheeling around; I guess that’s a little bit unsafe” (H6). Although this handler could recognize the danger, the other two participants with alert dogs had reduced cognitive ability and may not have been able to recognize the danger associated with leaving the care that carers provided before their dog was fully trained. 

#### 3.4.2. Immaturity of the Dog

A few of the ADs were perceived to be immature when acquired. Most participants became accustomed to this as the dogs matured and found their space within the family. Other individuals had more difficulty dealing with an immature dog. This was partly due to their expectations:

“Even now [6 months after acquiring a dog], and certainly for like the first three or four months, if [H6] left the house with her, there was an awful lot of work done by [H6] to look after [the dog], rather than [the dog] effectively looking after [H6], which I thought was going to be the effect”.

(P6)

This consequently increased the workload of the handler, who was already dealing with chronic and complex medical issues. This immaturity was perceived to considerably slow the process of integrating the dog into the handler’s life for this family, thereby delaying many benefits. 

## 4. Discussion

The challenges identified in these interviews provide new insight into the AD placement experience. As mentioned previously, all participants in this study reported receiving many benefits since acquiring their AD, and the importance of these should not be underestimated. We do not want to suggest that any person with a disability should be prevented from accessing an AD should this be considered as a possible strategy to ameliorate the impact of their disability, provided that the dog’s welfare can be ensured. However, the results reported in this paper identified factors that may cause challenges after an AD placement, such as: the handler’s medical condition/s, cognitive ability, social environment, and dog-related factors. It is evident that these factors are predominantly out of the handler’s control. Nonetheless, they considerably hindered the training and integration of the dog into the handler’s life, which negatively affected the handler-dog bond, the responsibility and independence of the handler, and potentially the dog’s short and long-term welfare. 

Variables associated with the handers’ disabilities, such as their medical condition and cognitive ability, appeared to be very prominent. Individuals with comorbid, complex or changing disability conditions experienced more challenges than those with relatively predictable disabilities. Additionally, these disability-related variables directly contributed to the extent to which other challenges were experienced, such as those associated with the social environment or dog factors. For example, handlers with complex conditions experienced extended hospital admissions in which the dog was unable to accompany them; those who had intellectual disability attended community day care programs and experienced more time away from their dog. This interfered with the dog’s ability to assist them, as the bond took longer to form and the dog subsequently experienced difficulties learning to perform desired functions, such as alerting to medical conditions. 

Similarly, handlers who lived in environments that were constantly changing, experienced more challenges than those in more predictable environments. Physical environmental changes contributed less than challenges due to the available social support in these environments. Typically, large social support networks are perceived to be more beneficial than having less social support [25]. In this study, however, individuals with small (typically informal) social support circles with very engaged members reported fewer challenges than those with many members who were constantly changing and had little investment in the handler. For example, individuals who employed carers or attended day programs had more formal support and experienced more challenges. Although these effects could be inherent to the nature of the disability, they did not facilitate integrating the dog into the handler’s life. For people with disabilities, various social support systems (formal vs informal) often do not communicate to coordinate support [26]. This brings challenges, as carers in these situations find it easier to do things for the handler or the dog instead of taking the time to learn to assist and implement the AD properly. This is similarly reported in other situations, where carers also find it easier to do things for the individual rather than assisting the handler to learn how to use a new form of assistive technology [27].

It is well established that the more disabilities an individual experiences, the more difficult it is for them to use traditional forms of assistive technology [27]. Individuals who experience mental health challenges or intellectual disabilities as part of their comorbid conditions are also known to use fewer forms of assistive technology compared to those with other types of disabilities [28]. This is consistent with the findings of the current study. Individuals with complex disabilities, mental health challenges or intellectual disabilities found it more difficult to effectively use an AD than individuals without these conditions. 

One might ask, therefore, why such people chose (or were advised) to acquire an AD. This is especially relevant since working with an AD is inherently complex compared to many other forms of assistive technology. One reason may be that these individuals and their families were desperate for assistance and had tried all other support options available to them [13]. Additionally, the handler or their family may have been told of the numerous benefits that the handler could receive, potentially without any discussion of the challenges that accompany working with an AD. That the handlers in this study did obtain considerable benefits from having an AD is not in question. Nonetheless, the challenges we observed contributed to reducing the benefits that were obtained and require careful consideration. 

### 4.1. Implications for Assistance Dog Organizations

The results emphasized in this paper, reinforce organizations’ need to comprehensively consider each prospective handler’s disabilities and abilities. More consideration may need to be given regarding their medical challenges, cognitive ability, their environment and the support that potential handlers have available to them. By focusing more energy into understanding these factors, organizations may be able to enhance vulnerable handlers’ experiences working with an AD. 

In addition, we suggest that organizations responsible for training and placing ADs should take time to consider the initial expectations handlers and members of their support network have prior to the placement of a dog, as the organization should work to minimize unrealistic expectations before they influence the perceived success of the relationship. Unrealistic expectations may contribute to perceived success and satisfaction with the dog, potentially preventing dogs from being returned due to behavioral problems and temperament issues, [29,30]. This is particularly important when working with first time handlers, who are least likely to have a realistic understanding of dogs’ abilities and behaviors. Dog selection is clearly always important and includes consideration for the ability and maturity of the dog, but again, this may be especially important for clients with more complex disabilities, who may be less able to deal with challenges, and for individuals who have not previously had any experience with an AD. 

The factors identified should be considered from the organizations’ perspective as well, as they contribute to the level of resources needed to be provided or the model used to integrate the dog into the handler’s life. We noted that the organizations represented in the current study were required to maintain contact with handlers with complex needs for months or years longer than what was required for those without these challenges, in order to ensure the dog was performing sufficiently and that it experienced good welfare. Several handers required many in-home visits and extensive ongoing support. In contrast, handlers without the challenges we identified commonly had only two follow-up visits with their organization after the initial placement period ended. For this reason, organizations supplying ADs may need to consider whether they have the resources and time available that complex cases require. They may need to consider altering their placement model, such as providing extensive initial training prior to the handler receiving the dog, or extra support throughout the placement period, perhaps for the life of the dog. This may be especially important for individuals who experience complex disabilities, mental health challenges and cognitive impairment.

With this in mind, we advise caution for the many organizations that are being set up to provide ADs to persons with a disability. It is critical that these organizations have expertise regarding a client’s specific disability and the effect of comorbid conditions. Some organizations already take these factors into consideration; some of our handlers reported that they were turned down by AD organizations because their case was too medically complex, or because the organization did not provide support for people with their specific medical condition or comorbid conditions. This may be appropriate, however it also makes it more difficult for these individuals to obtain an AD, and may result in their working with less experienced or less reputable organizations. Additional regulation is sorely needed worldwide in this field. Participating organizations have a responsibility to carefully consider whether an AD is the right form of assistive technology for a prospective recipient, and if they are the organization best suited to assist each person to achieve their goals. 

### 4.2. Future Directions

This paper draws on information provided by multiple types of individuals to obtain insight regarding the complexities that new handlers experience when working with an AD. The study included individuals who experienced a range of disabilities. However, not all disabilities that ADs have been trained to assist were represented and, therefore, future research should include other types of ADs as well. Additionally, only low participant numbers were able to be included, due to the intensive nature of the study. Due to time constraints, participants were interviewed at approximately six and twelve months after receiving their dog. Since ADs often work with a handler for approximately eight years [31], future research should look at expanding these time frames. 

## 5. Conclusions

This study explored the experiences of seven first-time AD handlers, six to twelve months after receiving their dog. Although many benefits were reported, as have been acknowledged previously, perceptions from family members, carers and AD instructors corroborated the handler’s reported challenges. This paper demonstrated that there are many factors, outside the handler’s control, that influence the challenges that they experience working with an AD. Organizations should consider these factors in relation to their clients and themselves to improve handler’s experiences and thus the dog’s welfare. 

## Figures and Tables

**Table 1:** Demographic information for case study participants.

Case Study	Type of AD	Handler Gender	Adult/Child	Participants	Code	Time of Interview (mo. Post AD Placement)	Mode of Interview
1	Seizure alert dog	Male	Young Adult	Handler	H1	6 12	In person In person
				Parent	P1	6 12	In person In person
				Instructor	ADI1	8	In person
2	Psychosocial AD	Male	Middle Age Adult	Handler	H2	6 12	In person In person
				Parent	P2	6 12	In person In person
				Carer	C2	7	Phone
				Instructor	ADI2	6 12	Phone In person
3	Mobility AD	Female	Middle Age Adult	Handler	H3	6 12	In person Phone
				Instructor	ADI3	6	In person
4	Medical alert dog	Male	Child (age 12)	Handler	H8	6	In person
				Parent	P8	6	In person
				Instructor	ADI8	6	In person
5	Guide dog	Male	Young Adult	Handler	H5	6 12	Phone Phone
				Parent	P5	6	Phone
6	Medical alert dog	Female	Young Adult	Handler	H6	6	In person
				Parent	P6	6 12	Phone Phone
				Other ^2^	O6	6	Phone
7	Guide dog	Female	Child (age 14) ^1^	Handler	H7	8 14	In person Phone
				Parent	P7	8	In person
				Instructor	ADI7	8	In person
				Other ^3^	O7	8	In person

^1^ the handler was 14 years old at 8 months, 15 years old at 14 months; ^2^ the AD organization’s psychologist; ^3^ the handler’s learning support educator at school.
