# Erratum: Gravrok, J., et al. Beyond the Benefits of Assistance Dogs: Exploring Challenges Experienced by First-Time Handlers. *Animals* 2019, *9*, 203

**DOI:** 10.3390/ani11010237

**Published:** 2021-01-19

**Authors:** Jennifer Gravrok, Dan Bendrups, Tiffani Howell, Pauleen Bennett

**Affiliations:** 1 Anthrozoology Research Group, School of Psychology and Public Health, La Trobe University, Edwards Rd, Flora Hill, VIC 3552, Australia; t.howell@latrobe.edu.au (T.H.); pauleen.bennett@latrobe.edu.au (P.B.); 2 Research Education and Development Team, Graduate Research School, La Trobe University, Edwards Rd, Flora Hill, VIC 3552, Australia; d.bendrups@latrobe.edu.au

The authors wish to make the following corrections [1]:

1. In Table 1, under case study 4, the code was originally labeled as H8, P8 and ADI 8; these labels should be H4, P4 and ADI 4, respectively. Please see the corrected Table 1 below. The codes were used correctly in the text.
2. In the first paragraph of 3.1.3 Hospital Admissions, the sentence “Even the handler who kept the dog with him during the hospital admission noted a regression in training.” should be removed. This was an error.
3. The authors apologize for any inconvenience caused.

## Figures and Tables

**Table 1:** Demographic information for case study participants.

Case Study	Type of AD	Handler Gender	Adult/Child	Participants	Code	Time of Interview (mo. Post AD Placement)	Mode of Interview
1	Seizure alert dog	Male	Young Adult	Handler	H1	6 12	In person In person
				Parent	P1	6 12	In person In person
				Instructor	ADI1	8	In person
2	Psychosocial AD	Male	Middle Age Adult	Handler	H2	6 12	In person In person
				Parent	P2	6 12	In person In person
				Carer	C2	7	Phone
				Instructor	ADI2	6 12	Phone In person
3	Mobility AD	Female	Adult	Handler	H3	6 12	In person Phone
				Instructor	ADI3	6	In person
4	Medical alert dog	Male	Child (age 12)	Handler	H4	6	In person
				Parent	P4	6	In person
				Instructor	ADI4	6	In person
5	Guide dog	Male	Young Adult	Handler	H5	6 12	Phone Phone
				Parent	P5	6	Phone
6	Medical alert dog	Female	Young Adult	Handler	H6	6	In person
				Parent	P6	6 12	Phone Phone
				Other ^2^	O6	6	Phone
7	Guide dog	Female	Child (age 14) ^1^	Handler	H7	8 14	In person Phone
				Parent	P7	8	In person
				Instructor	ADI7	8	In person
				Other ^3^	O7	8	In person

^1^ the handler was 14 years old at 8 months, 15 years old at 14 months; ^2^ the AD organization’s psychologist; ^3^ the handler’s learning support educator at school.
